# Functional Characterization of the Versatile *MYB* Gene Family Uncovered Their Important Roles in Plant Development and Responses to Drought and Waterlogging in Sesame

**DOI:** 10.3390/genes8120362

**Published:** 2017-12-12

**Authors:** Marie Ali Mmadi, Komivi Dossa, Linhai Wang, Rong Zhou, Yanyan Wang, Ndiaga Cisse, Mame Oureye Sy, Xiurong Zhang

**Affiliations:** 1 Oil Crops Research Institute of the Chinese Academy of Agricultural Sciences, Key Laboratory of Biology and Genetic Improvement of Oil Crops, Ministry of Agriculture, Wuhan 430062, China; alimarie85@hotmail.fr (M.A.M.); dossakomivi@gmail.com (K.D.); linhai827@163.com (L.W.); rongzzzzzz@126.com (R.Z.); 13664780477@163.com (Y.W.); 2 Centre d’Etudes Régional pour l’Amélioration de l’Adaptation à la Sécheresse (CERAAS), BP 3320, Thiès, Senegal; ncisse@refer.sn; 3 Laboratoire Campus de Biotechnologies Végétales, Département de Biologie Végétale, Faculté des Sciences et Techniques, Université Cheikh Anta Diop, 107000 Dakar, Senegal; oureyesy1@yahoo.fr

**Keywords:** abiotic stress, gene expression, *MYB*, plant growth and development, *Sesamum indicum*, transcription factors

## Abstract

The *MYB* gene family constitutes one of the largest transcription factors (TFs) modulating various biological processes in plants. Although genome-wide analysis of this gene family has been carried out in some species, only three *MYB* members have been functionally characterized heretofore in sesame (*Sesamum indicum* L.). Here, we identified a relatively high number (287) of sesame *MYB* genes (*SIMYBs*) with an uncommon overrepresentation of the 1R-subfamily. A total of 95% of *SIMYBs* was mapped unevenly onto the 16 linkage groups of the sesame genome with 55 *SIMYBs* tandemly duplicated. In addition, molecular characterization, gene structure, and evolutionary relationships of *SIMYBs* were established. Based on the close relationship between sesame and *Arabidopsis thaliana*, we uncovered that the functions of *SIMYBs* are highly diverse. A total of 65% of *SIMYBs* were commonly detected in five tissues, suggesting that they represent key TFs modulating sesame growth and development. Moreover, we found that *SIMYBs* regulate sesame responses to drought and waterlogging, which highlights the potential of *SIMYBs* towards improving stress tolerance in sesame. This work presents a comprehensive picture of the *MYB* gene family in sesame and paves the way for further functional validation of the members of this versatile gene family.

## 1. Introduction

Transcription factors (TFs) are important regulators of the expression of functional genes under different biological processes, including development, reproduction, and various environmental conditions [1]. Within the landscape of TFs that are present in the genome, the *MYB* family constitutes one of the largest and functionally diverse [2]. The MYB proteins are characterized by the MYB domain at the N-terminus which encodes 52 amino acid residues and contains one to four imperfect tandems repeats (R) [2]. According to the numbers of MYB repeat, *MYB* genes are classified into four different subfamilies, namely MYB –related proteins or 1R–MYB proteins (with 1R), R2R3–MYB proteins (with 2R), R1R2R3-MYB proteins (with 3R), and 4R-MYB proteins (with 4R) [3]. The R1R2R3-MYB proteins are found in animals, but the R2R3-MYB proteins are more frequent in plants [4]. The *MYB* TFs have been studied in diverse species and particularly in plants. Various numbers of *MYBs* have been detected suggesting that they have expanded to differing degrees: 177 *MYB* genes were found in *Citrus sinensis* [5], 231 in *Pyrus bretschneideri* [1], 475 in *Brassica rapa ssp. pekinensis* [6], 524 in *Gossypium hirsutum* [7], and 104 and 166 in *Lotus japonicas* and *Medicago truncatula*, respectively [8].

In plants, MYB proteins are key factors in physiological processes, shoot growth, root formation, organ development, metabolism, hormone signal transduction, and responses to biotic and abiotic stress tolerance [9,10,11,12,13]. For example, expression profiles analysis in *Arachis hypogaea* identified 30 *MYB* genes responsive to abiotic stress treatments [14]. Over-expression of *SpMYB* gene from *Solanum pimpinellifolium* improved abiotic and biotic stress resistance in tobacco [15]. A total of 44.67% and 47.21% *MYB* genes were found up- and down-regulated in *Arabidopsis* under cold stress, respectively [16]. Later on, Zhang et al. [17] reported the gene *TaLHY*, a 1R-*MYB* type as a positive regulator of resistance against stripe rust fungus and ear heading in *Triticum aestivum*. More recently, Xu et al. [18] demonstrated that the R2R3-type *MYB* gene *DcMYB6*, is involved in regulating anthocyanin biosynthesis in purple *Daucus carota* taproots. In the case of drought stress, many *MYB* genes have been isolated and demonstrated to be involved in drought responses in plants [19]. The transcriptional activation of cuticular wax biosynthesis by *MYB96* contributed to drought resistance in *Arabidopsis thaliana* [20]. A recent study revealed that *PbrMYB21* plays a positive role in drought tolerance by modulating polyamine synthesis through the regulation of the *ADC* expression in *Pyrus betulaefolia* [21]. Furthermore, Yin et al. [22] showed that *OsMYBR1* that was isolated from *Oryza sativa* improves drought tolerance of transgenic plants through the up-regulation of stress-related genes and the accumulation of osmoprotectants. Similarly, Butt et al. [23] demonstrated that *GaMYB85* confers good drought tolerance in *Gossypium arboreum*, most probably via an ABA-induced pathway. Altogether, these evidences demonstrated the versatility and importance of this gene family in plants.

Sesame (*Sesamum indicum* L.) is one of the oldest oilseed crops, which is widely grown in tropical and subtropical areas [24]. It has one of the highest oil contents (~55%) among major oilseed crops [25]. Drought and waterlogging represent the most important abiotic stresses that affect sesame plant growth and yield [26]. Sesame is a very sensitive crop to waterlogging [27]. The majority of sesame resources are susceptible to waterlogging damage at the different developmental stages [28], and so far, few studies have been conducted to unravel the genetic basis of waterlogging response in sesame [27,29,30]. In contrast, sesame is moderately tolerant to drought stress [31]. However, severe drought adversely impairs the plant normal growth and development [32], the reproduction and yield [26], and both the quality and quantity of the sesame oil as well [33,34,35]. Therefore, breeding sesame varieties with higher tolerance to these abiotic stresses is drawing great attention from breeders [36].

Given the potential roles of MYB proteins in the regulation of gene expression in response to environmental stresses, it is of the utmost interest to perform a genome-wide survey of this gene family in sesame. In this study, we identified at the genome-wide level 287 *MYB* genes and revealed that the MYB proteins are highly active in growth and adaptation to the major abiotic stresses namely drought and waterlogging in sesame. We also presented here a comprehensive analysis of the protein characteristics, gene classification and structure, chromosomal distribution, gene duplication, and phylogenetic relationships of sesame *MYB*s.

## 2. Materials and Methods

### 2.1. Identification and Sequence Analysis of MYB Proteins in Sesame

The sesame genome sequence was downloaded from Sinbase (http://ocri-genomics.org/Sinbase/) [37]. The HMM profile of MYB DNA-binding domain (PF00249) was downloaded from Pfam protein families database (http://pfam.sanger.ac.uk/) and was exploited for the identification of *MYB* genes from *Sesamum indicum* L. genome, using HMMER3 tool of the software Unipro UGENE (Unipro, Novosibirsk, Russia) [38]. To confirm the presence of MYB domain, the PROSITE (http://prosite.expasy.org/scanprosite/) and the SMART program (http://smart.embl-heidelberg.de/) [39] were used for further analysis. The isoelectric point and protein molecular weight of MYB proteins in sesame were obtained using ExPASy proteomics server (http://www.expasy.ch/tools/protparam.html). In addition, the sequences of 125 *Arabidopsis* R2R3-MYB proteins were downloaded from The Arabidopsis Information Resource (TAIR; http://www.arabidopsis.org/).

### 2.2. Chromosomal Location and Gene Duplication of MYB Genes in Sesame

The *MYB* genes were mapped onto the 16 linkage groups (LGs) of the sesame genome on the basis of their genomic location from Sinbase using the software MapChart 2.3 (Wageningen UR, Wageningen, Netherlands) [40]. The tandemly repeated *MYB* genes were identified when two sesame *MYB* genes were separated by three or fewer genes [41].

### 2.3. Phylogenetic Analysis and Functional Classification of MYB Proteins in Sesame

To characterize the evolutionary features of MYB proteins, we extracted all of the predicted MYB protein sequences from Sinbase (http://ocri-genomics.org/Sinbase/). According to the numbers of the conserved domains, we classified the sesame *MYB* genes into four subfamilies: R1-(single domain), R1R2-(2 domains), R1R2R3-(3 domains), and Atypical MYB (4 or more domains) [2]. Multiple alignments of their conserved domains were conducted using ClustalW program that was embedded in the MEGA6.0 software (Temple University, Philadelphia, PA, USA) [42], with a gap open penalty of 10 and gap extension penalty of 0.2. Thereafter, a Neighbor-Joining (NJ) tree was constructed with a 1000 bootstrap value. The tree was loaded along with the information of the gene CDS organization into the Gene Structure Display Server (GSDS2.0) (http://gsds.cbi.pku.edu.cn/) [43] to construct the exon-intron map.

Moreover, a Neighbour-Joining (NJ) tree was constructed from ClustalW aligned full-length of 259 R2R3-MYB proteins (125 MYB of *Arabidopsis* and 134 R2R3 MYB proteins for sesame) using MEGA6.0 software (Temple University, Philadelphia, PA, USA) [42] with the settings described above. Functional classification of the R2R3-*MYB* genes in sesame was performed according to their phylogenetic relationships with the corresponding *Arabidopsis AtMYB* genes.

To identify the orthologous genes of the sesame MYBs, we conducted a BLASTp search of their protein sequences against the protein database of *Arabidopsis* in TAIR. Hits with E-value ≤ 1^e−40^ and at least 70% similarity were considered significant [44]. The relationships between the orthologous genes of sesame and *Arabidopsis MYB*s were illustrated using Circos package [45].

### 2.4. Analysis of the Expression Patterns of Sesame MYB Genes in Different Tissues, under Drought and Waterlogging Stresses Using RNA-seq Data

To analyze the expression patterns of sesame *MYB* genes in various sesame tissues, we used RNA-seq data from root, stem seed, capsule, and leaf developed by our group and accessible from sesameFG (http://www.ncgr.ac.cn/SesameFG; [46]). Moreover, we investigated the expression levels of sesame *MYB* genes in responses to the two major abiotic stresses: waterlogging and drought. Transcriptomic data from a tolerant cultivar (Zhongzhi No. 13) under 3, 9, and 15 h of waterlogging stress were retrieved from GeneBank with the SRA accession number SRR2886790 [30]. Concerning the drought stress, the RNA-seq data of a drought tolerant genotype (ZZM0635) submitted to 3 (d_1_), 7 (d_2_) and 11 (d_3_) days of drought periods [47,48] were downloaded from http://www.ncbi.nlm.nih.gov/bioproject/PRJNA356988. The read per kilobase per million mapped reads (RPKM) values were log_2_ transformed and used for the construction of heatmaps with the MEV Software (J. Craig Venter Institute Inc., CA, USA) [49].

### 2.5. Plant Materials and Stress Treatments

To analyze the changes in expression of sesame *MYB* genes under drought and waterlogging stresses, two experiments were concomitantly conducted. The cultivars ZZM0635 and Zhongzhi No. 13, both obtained from the China National Genebank, Oil Crops Research Institute, Chinese Academy of Agricultural Sciences, were grown in pots for drought and waterlogging experiments, respectively. A three-days water withholding was applied to half of the seedlings when plants entered the early flowering stage with the soil volumetric water content falling from 35% to 10% following descriptions of Dossa et al. [47,48]. For the waterlogging application, the pots were flooded by standing in a plastic bucket filled with tap water to 3 cm above the soil surface and maintained for 9 h according to the experimental descriptions described by Wang et al. [30].

The roots of the seedlings were harvested after stress application for both stressed and control plants. The samples were harvested from three individual plants and were snap-frozen in liquid nitrogen and stored at −80 °C until further use.

### 2.6. RNA Isolation and Quantitative RT-PCR

Total RNAs from various tissues samples were extracted with Easy Spin RNA kit (Aidlab, Beijing, China). The quantity and quality of RNA samples were assessed by agarose gel electrophoresis and the spectrophotometer (Unico SpectroQuest Model SQ2800) measurement of the A260/A280 ratio. For cDNA synthesis, 1.5 μg of RNA was reverse transcribed using the Superscript III reverse transcription kit (Invitrogen, Carlsbad, CA, USA), according to the manufacturer’s instructions. Gene-specific primers were designed by using Primer5.0 [50]. We designed primers to amplify 29 *SIMYB* genes using Primer Premier 5.0 software (Premier Biosoft International, Palo Alto, CA, USA) (Table S5). The qRT-PCR was conducted on Roche Lightcyler^®^ 480 instrument using SYBR Green Master Mix (Vazyme), according to the manufacturer’s protocol. We performed each reaction using a 20 µL mixture containing 10 µL of 2xChamQ SYBR qPCR Master Mix, 8 µL of nuclease-free water, 2.4 µL of each primer (10μM), and 2 µL of diluted cDNA. All of the reactions were run in 96-well plates and each cDNA was analyzed in triplicate. The reactions were performed with the following cycling profile: 95 °C for 30 s, followed by 40 cycles of 95 °C/10 s, 60 °C/30 s. Each reaction was performed in biological triplicates, and the relative gene expression values were calculated using the 2^−ΔΔCt^ method, as described by [51]. The sesame *Actin* gene (*SIN_1006268*) was used as the internal reference gene [52]. For stress related genes, they were considered significantly up- and down-regulated when their relative expression fold change (stress/control) were >2 and <0.5, respectively [53].

## 3. Results

### 3.1. Identification of Sesame MYB Genes and Analysis of Their Sequence Features

We identified in the sesame genome, 287 sequences that contain MYB or MYB-like repeats. We followed the united nomenclature for *Sesamum indicum* genes and used *SIMYB* (for *S*. *indicum MYB*) to represent *MYB* genes from sesame.

All of the putative *MYB* genes were further confirmed by SMART and PROSITE searches for the presence of the two MYB repeats. Finally, we confirmed 287 typical *MYB* genes in the whole genome of sesame, including 134 R2R3-*MYB*, 5 R1R2R3-*MYB*, 147 *MYB* related, and 1 atypical *MYB* genes. The *MYB* related genes represent more than half of the total *MYB* genes, and thus constitute the largest subfamily of *MYB* genes in sesame (Table 1).

The isoelectric point (pI) values for SIMYB proteins ranged from 4.73 (SIMYB3) to 9.91 (SIMYB240). The molecular weight (Mw) was from 9208.63 kDa (SIMYB22) to 73269.43 kDa (SIMYB18) (Table S1).

### 3.2. Chromosomal Distribution and Duplication Events among Sesame MYB Genes

**Figure 1:**
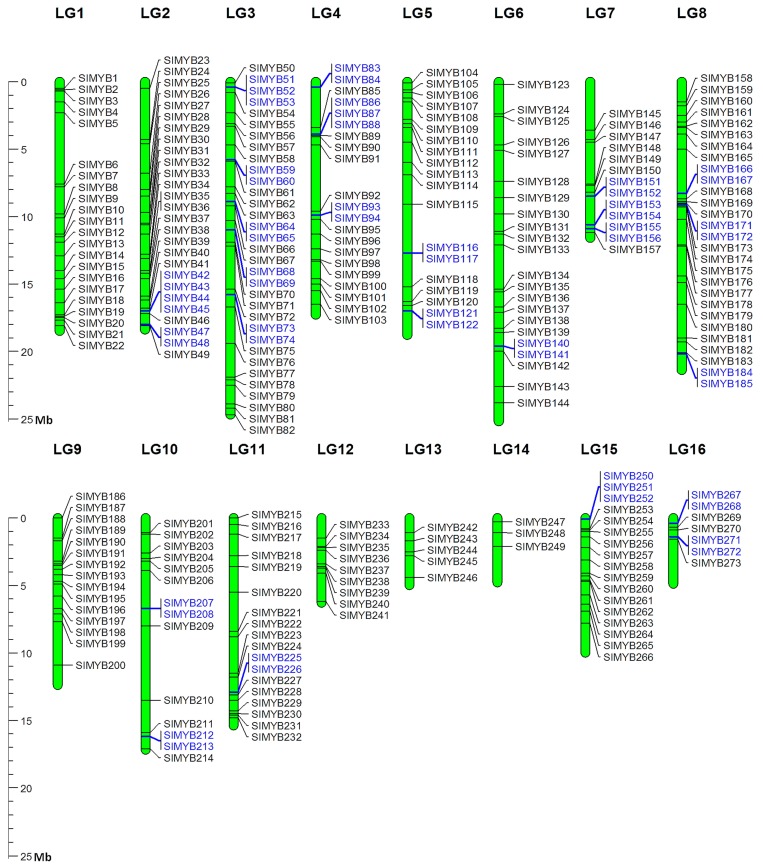
Mapping of sesame *MYB* genes based on their physical positions. Vertical green bars represent linkage groups (LG) of the sesame genome. The LG numbers are indicated at the top of each LG. The genes colored in blue are the tandemly duplicated *SIMYBs*. The scale on the left is in megabases (Mb).

Genome chromosomal location analyses showed that the sesame *MYB* genes were distributed throughout all of the 16 linkage groups (LG) (Figure 1). In the currently released genome sequences, 273 *SIMYB* genes (95% of the total *SIMYBs*) were successfully mapped onto the LGs, whereas 14 genes were remained on as yet unmapped scaffolds. All of the *SIMYB* genes were named from *SIMYB1* to *SIMYB287* following their order on the LGs and unanchored sequences. The anchored *SIMYB* genes were unevenly distributed onto the 16 LGs of the sesame genome. The LG03 encompassed the largest number of *SIMYBs* (33 genes), followed by 28 genes on LG08. In contrast, only three *SIMYB* genes were detected on LG14.

We detected 55 genes (20% of the anchored *SIMYBs*) that were involved in tandem duplicated arrays with sizes ranging from two to four *SIMYBs* on the LG02, LG03, LG04, LG05, LG06, LG07, LG08, LG10, LG11, LG15, and LG16 (Figure 1).

### 3.3. Phylogenetic Relationships, Classification and Exon-Intron Structures of SIMYB Gene Family in Sesame

**Figure 2:**
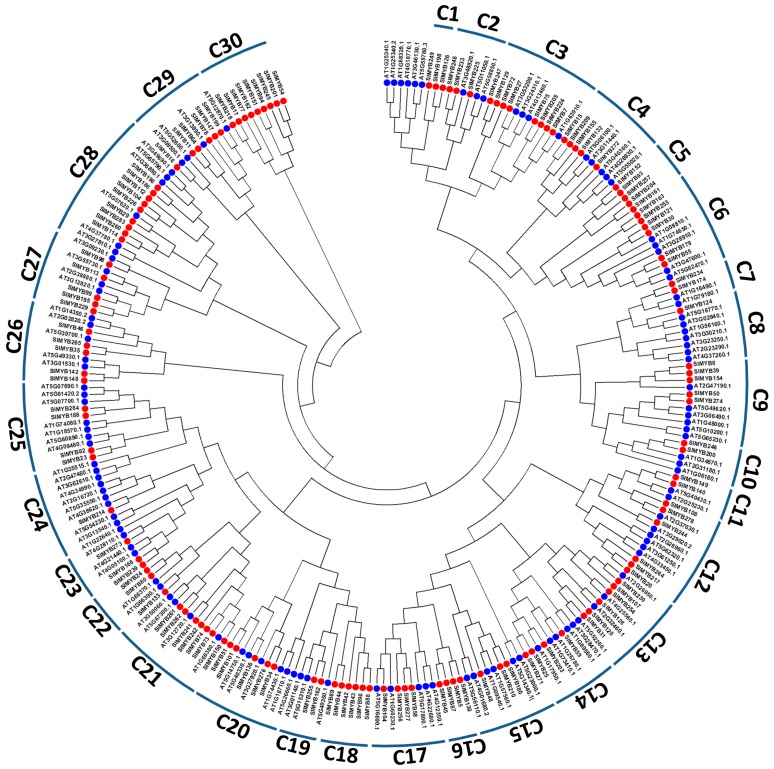
Phylogenetic relationships of the R2R3-MYB subfamily between sesame and *Arabidopsis*. The subgroups were designated as C1 to C30 following the classification in *Arabidopsis* [2]. Sesame *SIMYBs* are represented by the red dot whereas *Arabidopsis MYB* are labeled by a blue dot.

Using the *Arabidopsis* R2R3-MYB proteins as reference for classification, we subdivided the *SIMYB* members into 30 subgroups (designated as C1–C30 in this study) according to the sequence similarity and topology (Figure 2; Table S2). Most of the subgroups in our classification were in accordance with those in *Arabidopsis*, and only seven subgroups (C1, C5, C11, C16, C18, C29, and C30) were not retrieved in the phylogenetic tree of *Arabidopsis* MYB proteins. According to the functions described for *Arabidopsis* R2R3-MYB subgroups [2], we therefore inferred the functions of the corresponding subgroups of R2R3-MYB in sesame (Table S2).

**Figure 3:**
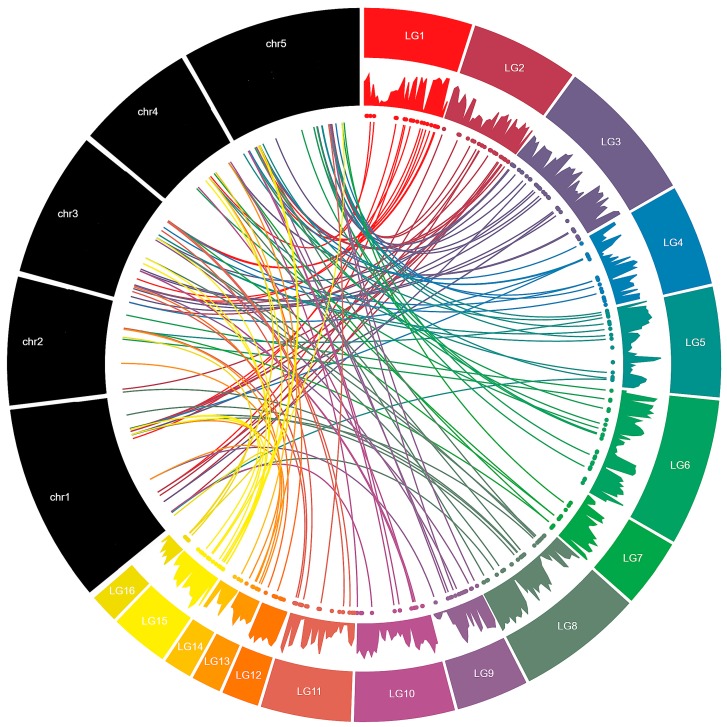
Syntenic relationships between *MYB* genes in sesame and *Arabidopsis* genomes. The black bars represent the chromosomes of *Arabidopsis* whereas the colored bars represent the linkage groups of the sesame genome. The numbers LG01–16 represent linkage groups within the sesame genome and the five *Arabidopsis* chromosomes are labeled Chr1–Chr5. The area chart displays the gene density along the LGs in the sesame genome. The colored dots represent the locations of the *SIMYB* genes along the LGs. Colored lines indicate orthologous genes in sesame and *Arabidopsis*.

Synteny analysis of *SIMYB* genes when compared to those of *Arabidopsis* could provide more functional insight. Comparative orthologous mapping of *MYB* genes between the two species revealed that 133 *SIMYBs* are orthologs to *Arabidopsis MYB* genes across the 16 LGs of the sesame genome. The LG03 and LG02 exhibited the highest numbers of orthologous genes (16 and 15 gene pairs, respectively) while only one gene pair was detected on LG14. Moreover, *Arabidopsis MYB* genes retained from 1 to 6 copies of *SIMYBs* while conversely, no gene in sesame has conserved more than a single copy in *Arabidopsis* genome (Figure 3). Based on the functions described for the *Arabidopsis MYB* genes [1,2], we assigned various functions to the orthologous *SIMYBs* (Table S3).

The exon/intron structure analysis for the 287 *SIMYBs* indicated that most of the coding sequences were disrupted by introns, with exception of 16 genes (Figure S1). The numbers of exon of the 287 *SIMYB* genes ranged from 1 to 31. In addition, we identified five genes that were possessing high numbers of exons from 16 to 31 (*SIMYB70*, *SIMYB83*, *SIMYB187*, *SIMYB197,* and *SIMYB135*). The clustering pattern of *SIMYBs* was not obviously consistent with the exon/intron structures. However, in few cases, some of the genes in the same cluster exhibited a similar exon/intron structure. For example, the intronless genes *SIMYB119*, *SIMYB32*, *SIMYB77*, *SIMYB190,* and *SIMYB182*, all clustered together on the phylogenetic tree. Moreover, clustering patterns of *SIMYB* genes did not strictly follow their subfamily’s classification (Figure S1). 

### 3.4. Expression Patterns of SIMYB Genes in Different Tissues of Sesame

**Figure 4:**
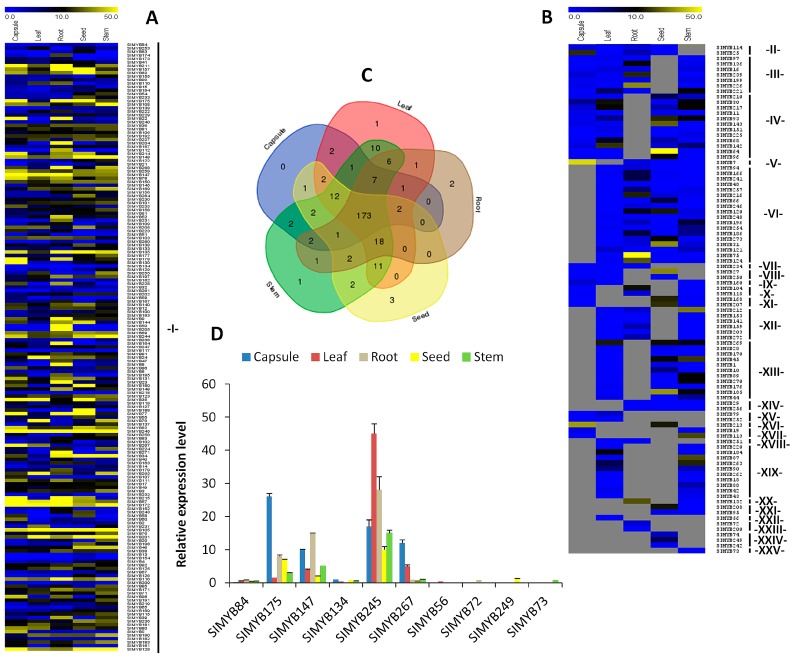
Expression profile analysis of *MYB* genes in five sesame tissues. The *SIMYB* genes expressed (RPKM > 1) in leaf, stem, root, seed and capsule were represented according to their tissue specificity: (**A**) *SIMYB*s commonly expressed in the five tissues; (**B**) *SIMYBs* expressed in only some tissues; (**C**) Venn diagram exhibiting the numbers of common and unique *SIMYB* genes expressed in the various sesame tissues; and, (**D**) Validation through qRT-PCR of the expression patterns of ten selected genes. Gray cells represent genes not expressed. -I- to -XXV- represent the groups of genes according to their tissue specificity (Table S4).

The gene expression pattern can provide important clues for its function. To explore the expression pattern of *MYB* gene family in sesame development and growth, we investigated the relative expression level of *SIMYBs* in five tissues, including root, capsule, leaf, stem, and seed. The results revealed that *SIMYB* genes have a variety of expression patterns in different tissues of sesame (Figure 4), and could be classified into 25 groups (I to XXV) according to their tissue-specificity expression (Table S4). A total of 268 *SIMYB* genes were expressed (RPKM > 1) in the different tissues, although their transcript abundance was highly varied according to the tissues. Of these expressed genes, 173 *SIMYBs* (~65%) were commonly detected in all of the tissues, suggesting that they regulate sesame development and growth (Figure 4a). We found some highly expressed genes (*SIMYB69*, *SIMYB106*, *SIMYB171, SIMYB175, SIMYB245*, etc.) in the tissues, some low expressed genes (*SIMYB84*, *SIMYB134*, etc.), and also found some tissue-specific *SIMYBs*. For instance, the gene *SIMYB73* was only expressed in the stem; two genes, including *SIMYB72* and *SIMYB200* were exclusively expressed in the sesame root; one gene in the leaf (*SIMYB56*), three genes (*SIMYB74*, *SIMYB242,* and *SIMYB249*) were expressed in the seed and one gene (*SIMYB19*) was exclusively expressed in the capsule (Figure 4b,c; Table S4). To further validate the gene expression profile from RNA-seq data, we performed qRT-PCR analysis with ten selected genes, including six genes with high or low expression levels from the group I and one tissue-specific gene each from the groups XXII, XXIII, XIV, and XXV. For all of the genes tested, qRT-PCR results confirmed well the RNA-seq expression profiles, proving the accuracy of the RNA-seq dataset that was used in this study (Figure 4d).

### 3.5. Expression Profiles of SIMYB Genes in Response to Waterlogging Stress

**Figure 5:**
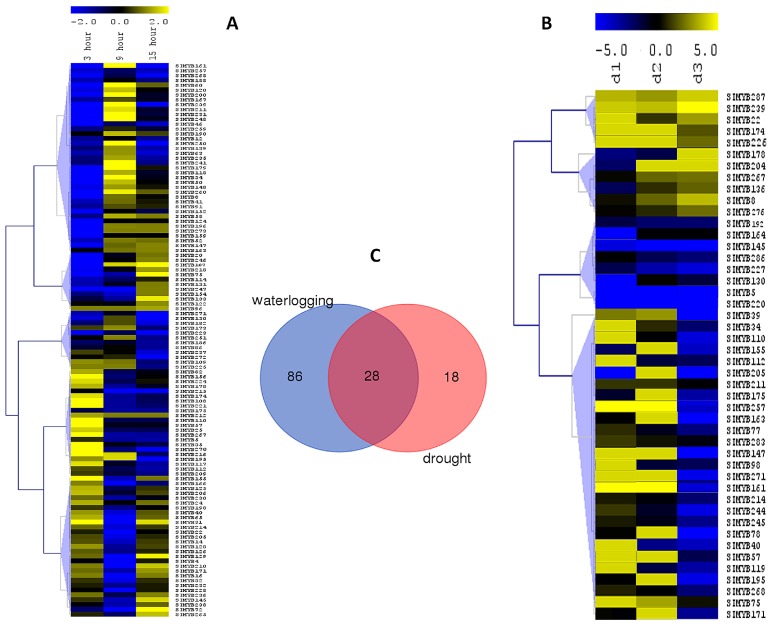
Transcriptome profiling of *MYB* gene expression under waterlogging and drought stress. (**A**) Heatmap displaying expression changes of differentially expressed *MYB* genes in sesame plants stressed for 3 h, 9 h and 15 h of waterlogging compared with the control; (**B**) Heatmap displaying expression changes of differentially expressed *MYB* genes in sesame plants stressed for 3 days (d_1_), 7 days (d_2_), and 11 days (d_3_) of drought compared with the control; and, (**C**) Venn diagram depicting the shared and stress-specific differentially expressed genes between waterlogging and drought.

**Figure 6:**
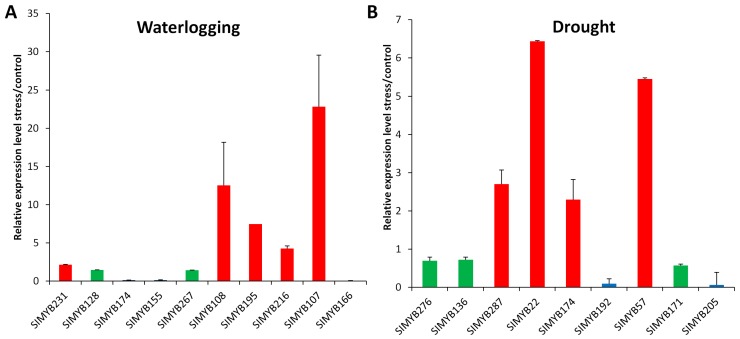
*MYB* gene induction rates in sesame roots during abiotic stress treatments. (**A**) 9 h of waterlogging; (**B**) 3 days of drought. Transcripts abundance was quantified through qRT-PCR and the experimental values were normalized using sesame *Actin* as reference gene. The histograms represent the relative expression values of induction rates (stress/control). The green bars represent the no significantly regulated genes, the red bars represent the genes significantly up-regulated (induction rate > 2) and the blue bars represent the significantly down-regulated genes (induction rate < 2).

The expression of *SIMYB* genes under waterlogging stress was examined at 3, 9, and 15 h after treatment. In total, we identified 114 *SIMYBs* as differentially expressed genes (DEGs) with various expression patterns across the three time points of waterlogging stress application (Figure 5a). The observed expression patterns revealed that *SIMYB* genes could be categorized into five main groups. The group I was composed of 39 genes, which were strongly induced at 9 h of stress application. The group II gathered together 12 genes which were induced when the stress became more intense (15 h). The group III encompassed 12 genes induced at 3 and 9 h, but their expression levels were significantly decreased at 15 h under stress. The group IV included 22 genes that were up-regulated at the beginning of the stress application (3 h), but their expression levels decreased with an increasing level of stress. Finally, the group V was composed of 29 down-regulated genes with their expression levels significantly down-regulated at 9 h under stress but induced at the beginning of the stress (3 h) and under severe stress (15 h). The RNA-seq data was further verified by qRT-PCR experiment which was performed on root samples of a waterlogging-tolerant cultivar Zhongzhi No.13 based on ten selected *SIMYB* genes differentially expressed (Figure 6a). A total of five genes were detected up-regulated with the gene *SIMYB107*, the most induced one (>22-fold increase in gene expression). In contrast, the genes *SIMYB166*, *SIMYB155,* and *SIMYB174* were down-regulated under waterlogging, as revealed by the RNA-seq data.

### 3.6. Expression Profiles of SIMYB Genes in Response to Drought Stress 

Previously developed RNA-seq data under drought stress [42,43] allowed for us to analyze the expression profiles of *SIMYBs* under this important abiotic stress. First, we retrieved all *SIMYB*s from the DEGs at d_1_, d_2_, and d_3_ when compared to control (period prior to water stress application). A total of 46 *SIMYBs* were found as DEGs across the different drought application periods, and their expression profiles were subsequently summarized in the heatmap represented in the Figure 5b.

The results showed that *SIMYB*s regulating drought responses could be assembled into four clusters: The first group (I) represented five genes constitutively up-regulated under drought stress with a striking induction at d_3_. The group II gathered together six genes with increased activity under severe stresses (d_2_ and d_3_). The group III was composed of eight genes constitutively down-regulated under drought stress. Finally, the group IV encompassed 27 genes highly induced at d_1_ or d_2_, but down-regulated under severe drought stress (d_3_).

To further confirm the transcriptomic expression patterns of *SIMYBs*, we selected nine *SIMYBs* and performed qRT-PCR analysis in the drought tolerant genotype after three days of drought stress. Among these genes, we found four *SIMYBs,* including *SIMYB22*, *SIMYB57*, *SIMYB174,* and *SIMYB287*, which were significantly up-regulated in response to drought, with *SIMYB22* being the most induced gene. The genes *SIMYB192* and *SIMYB205,* in contrast, were down-regulated in response to drought stress exactly, as shown in the RNA-seq results. However, we found three *SIMYB* genes (*SIMYB136*, *SIMB171,* and *SIMYB267*) that were not significantly regulated under drought stress, as their expression levels in the stressed plants when compared to the control ones remained unaffected (Figure 6b).

By crossing the significantly regulated *SIMYB* genes under drought and waterlogging stresses, we found that a total of 132 genes (46% of the total *SIMYB* genes) were implicated in these major abiotic stresses in sesame. Moreover, we identified some stress-specific genes while 28 genes were commonly involved in drought and waterlogging stresses (Figure 5c). Most of these shared genes displayed contrasting expression patterns under both stresses. For example, the gene *SIMYB174* was highly down-regulated under waterlogging, but was found strongly up-regulated under drought stress (Figure 6).

## 4. Discussion

The function of *MYB* genes has been extensively investigated in numerous plants species, but, to date, no comprehensive analysis of the *MYB* gene family members has been reported in sesame and their functions are still largely unknown. In this study, we comprehensively studied this important gene family based on bioinformatic tools and expression profiling. We identified 287 *SIMYB* genes, which is quite higher than the 198 *MYB* genes reported in *Arabidopsis*, 183 in rice [16], 247 in soybean [54], 177 in sweet orange [5], 125 in *Jatropha curcas* [55], 231 in *Pyrus bretschneideri* [1], and 104 and 166 in *Lotus japonicus* and *Medicago truncatula*, respectively [8]. To date, only two species, namely, cotton and Chinese cabbage had much higher *MYB* genes than sesame, which could be attributed to their bigger genome sizes [6,7]. Hence, according to the previous reports related to the expansion of gene families in sesame [44,53,56,57], we speculated that sesame has retained and expanded during the evolutionary process, the *MYB* family members that may play essential roles in various biological processes. Similarly as reported in cotton [7], soybean [54], rice, and *Arabidopsis* [16], tandem duplication events have contributed to the expansion of the *MYB* gene family in sesame. More importantly, we deduced that tandem duplication events have also contributed to the functional divergence of *SIMYBs* since some tandem duplicated *SIMYB* genes were predicted distinct functions.

One surprising feature of the *SIMYB* gene family is the over-representation of the 1R-MYB subfamily. The 2R-MYB subfamily has been reported to be the largest subfamily of MYB family, followed by the 1R-MYB in many plant species [7,16,58,59]. Contrarily to the 2R-MYB, the 1R-MYB subfamily is less-studied and their functions were assigned for circadian and light regulation, cell differentiation, and telomeric DNA binding [9]. In sesame, we have found its members to be highly active in abiotic stress response, suggesting a novel role of this subfamily. Nonetheless, the abundance of 1R-MYB subfamily members in the sesame genome is intriguing and may deserve in-depth functional investigations.

To date, only three *MYB* genes (*SIMYB186* (*SIN_1010473*), *SIMYB261* (*SIN_1025617*), and *SIMYB257* (*SIN_1004921*)) have been functionally characterized as being involved in the corolla and petiole pigmentation in sesame [52]. Wherefore, revealing the functions of the members of this important gene family should be the focus of future studies in sesame. The R2R3-*MYB* genes are known to be involved in plant specific processes, such as control of secondary metabolism or cellular morphogenesis, defense, pigmentation, and root formation [60]. Structurally similar MYB proteins within species were found to be functionally orthologous. For example, the genes *ZmMYBC1*, *ZmMYBPL* from maize and *PhMYBAN2* from *Petunia* are structurally related and play the same function: control of anthocyanin synthesis [7]. Hence, we performed an evolutionary relationship analysis of the R2R3-MYB subfamily in sesame when compared with the well-studied species *Arabidopsis*. Out of 30 sesame clades, 23 clades were consistent with those in *Arabidopsis* [1,2], providing an excellent reference to explore the functions of sesame R2R3-*MYB* genes [61]. For example, *SIMYB50*, *SIMYB74*, *SIMYB242,* and *SMYB261* were assembled together with *Arabidopsis AtMYB044* and *AtMYB077* into the clade 21, referring to abiotic stress response and anthocyanin biosynthesis [62]. The genes *SIMYB24* and *SIMYB80* were grouped into the clade 22 with two *Arabidopsis* genes *AtMYB90* and *AtMYB113,* representing the functional clade of anthocyanin biosynthesis [63]. The genes *SIMYB72* and *SIMYB200* were found in the groups C3 and C10, respectively, related to root development. Accordingly, based on tissue RNA-seq analysis, we uncovered that these genes were exclusively expressed in sesame roots. Seven R2R3-*SIMYB* subgroups of sesame have no representative in *Arabidopsis,* suggesting that these proteins might have specialized roles that were either lost in *Arabidopsis* or gained after divergence from the last common ancestor [2]. Furthermore, the close relationship between sesame and *Arabidopsis* helped to identify some homolog *MYB* genes and allowed for us to predict the functions of *SIMYB* genes [53].

Few *MYB* genes have been reported to be involved in plant developmental processes [6]. In sesame, 65% of *SIMYB* genes were expressed in all of the tissues, indicating that these genes might contribute in various aspects of growth and developmental processes. In contrast, we detected few tissue-specific *SIMYBs* that may play major role in their respective tissues [2]. To cite an instance, the gene *SIMYB200,* which is exclusively expressed in the sesame root, is the homolog of the gene *AtMYB93* which regulates lateral root development in *Arabidopsis* [64]. Until now, the sesame root system architecture has not been yet examined at the molecular level [36]. Therefore, *SIMYB200* could be a potential gene to be targeted for functional characterization in the sesame root. In addition, the homolog of the sesame seed-specific genes *SIMYB74*, *SIMYB242,* and *SIMYB249* in *Arabidopsis* (*AtMYB113*) was found to be involved in anthocyanin biosynthesis [65]. We suspected that these genes may be involved in seed coat coloration in sesame.

Waterlogging is a common adverse environmental condition that limits sesame plant growth and reproduction [30]. Similarly, drought stress constitutes the second major abiotic stress in sesame production [26]. Unfortunately, advances in developing abiotic stress tolerant sesame varieties have been hampered by the lack of functional gene resources [36]. Increasing evidences have shown that *MYB* TFs are implicated in drought response in various plant species, including *Arabidopsis thaliana*, *Zea mays*, *Gossypium herbaceum*, *Vitis vinifera*, *Oryza sativa*, *Solanum tuberosum*, *Triticum aestivum,* and *Glycine max* [66,67,68,69,70,71,72]. In sesame, 16% of the total *SIMYB* genes were significantly active in drought stress responses, and most of their homologs in *Arabidopsis* are described as abiotic stress responsive genes. By way of illustration, the homologs of the sesame drought responsive genes *SIMYB204* (*AtMYB015*), *SIMYB77*, *SIMYB245*, *SIMYB78* (*AtMYB073*), and *SIMYB8* (*AtMYB2*) were proved to be implicated in drought responses in *Arabidopsis*. This suggests a conservation of gene function across the two species, implying that these identified genes could be regarded as candidate gene resources for drought tolerance improvement in sesame [1,2].

On the other hand, little is known concerning the role of MYB genes in the waterlogging response in plants. It was shown that *AtMYB2* is induced by hypoxia, with mRNA levels peaking after 2–4 h under hypoxic conditions [73]. The gene *TaMyb1* (ortholog of *AtMYB2*) exhibited a dramatic increase in transcripts under waterlogging stress, indicating its positive involvement in alleviating the damages in common wheat root [74]. The gene *SIMYB8,* which is the homolog of *AtMYB2,* was also highly induced after 9 h waterlogging stress in sesame, indicating a functional conservation. Recently, based on a transcriptome profiling under waterlogging stress in kiwifruit plants, Zhang et al. [75] found that the *MYB* TFs were abundant in the differentially expressed genes. In the present study, 40% of *SIMYBs* was significantly associated with waterlogging stress responses, representing potential genes for enhancing sesame endurance under this important abiotic stress. The higher number of *SIMYBs* regulated under waterlogging as compared to drought confirms sesame’s greater susceptibility to waterlogging stress [30].

*SIMYBs* commonly involved in drought and waterlogging responses are of a great interest as they could be targeted for improvement towards tolerance to both stresses simultaneously. Our results are in agreements with previous reports which showed that some *MYB* gene members are key regulators of several abiotic stresses, including cold, osmotic stress, salt, etc. [76,77,78]. However, it appears that *SIMYBs* may function differentially under drought and waterlogging stresses as shown by their contrasting expression patterns under these stresses. Therefore, in-depth analysis of these particular genes is needed to get insight into the mechanisms of co-regulation of drought and waterlogging stresses in sesame.

## 5. Conclusions

In the present study, we analyzed the characteristics of *MYB* gene structures, phylogenetic relationships, chromosomal locations, and their functional diversification in sesame. For the first time in sesame, we identified 287 *SIMYB* genes that were unevenly distributed on the 16 LGs. *SIMYBs* are quite abundant in sesame probably due to tandem duplication events with an over-representation of the 1R-MYB subfamily. Phylogenetic-based prediction of the *SIMYB* functions indicated that they are involved in diverse biological processes. Expression profiling in various tissues suggested that *SIMYBs* are highly active in modulating sesame growth and development. Moreover, by integrating RNA-seq data and qRT-PCR analysis, we demonstrated that *SIMYBs* are key transcription factors regulating sesame responses to drought and waterlogging stresses. Altogether, we provide here a firm groundwork for the future functional characterization of *SIMYBs*.

## Figures and Tables

**Table 1:** Classification of the *MYB* gene family and distribution across the linkage groups (LG) of the sesame genome.

Linkage Groups	R1-MYB	R1R2-MYB	R1R2R3-MYB	Atypical-MYB	Total
**LG01**	12	8	1	1	22
**LG02**	11	16			27
**LG03**	17	15	1		33
**LG04**	12	9			21
**LG05**	10	8	1		19
**LG06**	11	10	1		22
**LG07**	5	8			13
**LG08**	19	9			28
**LG09**	8	7			15
**LG10**	7	7			14
**LG11**	10	7			17
**LG12**	6	4			10
**LG13**	1	4			5
**LG14**	1	3			4
**LG15**	7	10			17
**LG16**	4	3			7
**Scaffold**	6	6	1		13
**Total**	147	134	5	1	287
**Percentage (%)**	51.22	46.69	1.74	0.35	

## Supplementary Materials

The following are available online at www.mdpi.com/2073-4425/8/12/362/s1. Table S1: Full details of the sesame *SIMYB* genes, subfamily, ORF length, number of CDS, genome location and protein characteristics. Table S2: Classification and functional prediction of R2R3- *SIMYB* genes based on *Arabidopsis* functional clades [1,2,79,80,81,82,83,84,85,86,87,88,89,90,91,92,93,94,95,96,97,98,99]. Table S3: Function prediction of *SIMYBs* based on sesame and *Arabidopsis* orthology. Table S4: List of the *SIMYB* genes expressed in five tissues of sesame. Table S5: List of gene specific primers used for qRT-PCR expression analysis of *MYB* genes in sesame under drought, waterlogging and various tissues. Figure S1: Gene structures of MYB proteins detected in sesame. Exon and intron are represented by blue box and black line, respectively.
